# Supplementary material for: From sequence to enzyme mechanism using multi-label machine learning
Source: BMC Bioinformatics. 2014 May 19;15:150. doi: 10.1186/1471-2105-15-150 (PMC4229970; doi:10.1186/1471-2105-15-150)
Supplement: Additional file 2 — Java code of ml2db. Additional file ml2db_code.tar.gz contains the Java source code to run the multi-label machine learning experiments and save the results to database. The code’s Javadoc is included. [file 1471-2105-15-150-S2.zip › additional file 2/ml2db/ml2db/doc/mulan/classifier/neural/DataPair.html]

DataPair


JavaScript is disabled on your browser.


- Overview
- Package
- Class
- Use
- Tree
- Deprecated
- Index
- Help

- Prev Class
- Next Class

- Frames
- No Frames

- All Classes

- Summary:
- Nested |
- Field |
- Constr |
- Method

- Detail:
- Field |
- Constr |
- Method


mulan.classifier.neural

## Class DataPair

- java.lang.Object
- - mulan.classifier.neural.DataPair

- ---

    

  ```
  public class DataPair
  extends java.lang.Object
  ```

  Class for representation of a data-pair instance. The data pair contains
  an input pattern and respected true or expected output pattern for the input pattern.

  Version:
  :   2012.02.27

  Author:
  :   Jozef Vilcek

- - ### Constructor Summary

    Constructors

    Constructor and Description

    `DataPair(double[] inputPattern,
    double[] trueOutput)`

    Creates a `DataPair` instance.
  - ### Method Summary

    Methods

    | Modifier and Type | Method and Description |
    | --- | --- |
    | `static java.util.List<DataPair>` | `createDataPairs(MultiLabelInstances mlDataSet, boolean bipolarOutput)` Creates a `DataPair` representation for each `Instance` contained in `MultiLabelInstances` data set. |
    | `double[]` | `getInput()` Gets the input pattern. |
    | `double[]` | `getOutput()` Gets the ideal/expected output pattern. |
    | `boolean[]` | `getOutputBoolean()` Gets the ideal/expected output pattern as boolean values. |

    - ### Methods inherited from class java.lang.Object

      `equals, getClass, hashCode, notify, notifyAll, toString, wait, wait, wait`

- - ### Constructor Detail


    - #### DataPair

      ```
      public DataPair(double[] inputPattern,
              double[] trueOutput)
      ```

      Creates a `DataPair` instance.

      Parameters:
      :   `inputPattern` - the input pattern
      :   `trueOutput` - the true/expected output pattern for the input
  - ### Method Detail


    - #### getInput

      ```
      public double[] getInput()
      ```

      Gets the input pattern.

      Returns:
      :   the input pattern


    - #### getOutput

      ```
      public double[] getOutput()
      ```

      Gets the ideal/expected output pattern.

      Returns:
      :   the output pattern


    - #### getOutputBoolean

      ```
      public boolean[] getOutputBoolean()
      ```

      Gets the ideal/expected output pattern as boolean values.
      This is useful when output represents labels bipartition.
      If output values in `double[]` are not in boolean representation,
      then output of this method is might not be valid.
      The computation is as follows:  
      - if value is equal to 1, then output is `true` in boolean  
      - if value is other than 1, then output is `false` in boolean

      Returns:
      :   the boolean representation of the output pattern


    - #### createDataPairs

      ```
      public static java.util.List<DataPair> createDataPairs(MultiLabelInstances mlDataSet,
                                             boolean bipolarOutput)
      ```

      Creates a `DataPair` representation for each `Instance` contained in
      `MultiLabelInstances` data set. The `DataPair` is a light weight representation
      of instance values (by double values), which is useful when iteration over the data and its
      values.

      Parameters:
      :   `mlDataSet` - the `MultiLabelInstances` which content has to be
          converted to list of `DataPair`
      :   `bipolarOutput` - indicates whether output values should be converted
          to bipolar values, or left intact as binary

      Returns:
      :   the list of data pairs


- Overview
- Package
- Class
- Use
- Tree
- Deprecated
- Index
- Help

- Prev Class
- Next Class

- Frames
- No Frames

- All Classes

- Summary:
- Nested |
- Field |
- Constr |
- Method

- Detail:
- Field |
- Constr |
- Method
